# Comparative Proteomics at the Critical Node of Vigor Loss in Wheat Seeds Differing in Storability

**DOI:** 10.3389/fpls.2021.707184

**Published:** 2021-08-30

**Authors:** Xiuling Chen, Andreas Börner, Xia Xin, Manuela Nagel, Juanjuan He, Jisheng Li, Na Li, Xinxiong Lu, Guangkun Yin

**Affiliations:** ^1^National Crop Genebank, Institute of Crop Science, Chinese Academy of Agricultural Sciences, Beijing, China; ^2^Applied Technology Research and Development Center for Sericulture and Special Local Products of Hebei Universities, Institute of Sericulture, Chengde Medical University, Chengde, China; ^3^Department of Genebank, Leibniz Institute of Plant Genetics and Crop Plant Research, Gatersleben, Germany

**Keywords:** wheat, seed longevity, long-term storage, differentially accumulated proteins, artificial aging

## Abstract

The critical node (CN, 85% germination) of seed viability is an important threshold for seed regeneration decisions after long-term conservation. Dependent on the germplasm, the storage period until CN is reached varies and information on the divergence of the proteomic profiles is limited. Therefore, the study aims to identify key proteins and mechanisms relevant for a long plateau phase and a late CN during artificial seed aging of wheat. Seeds of the storage-tolerant genotype (ST) TRI 23248, and the storage-sensitive genotype (SS) TRI 10230 were exposed to artificial ageing (AA) and extracted embryos of imbibed seeds were analyzed using an iTRAQ-based proteomic technique. ST and SS required AA for 24 and 18 days to reach the CN, respectively. Fifty-seven and 165 differentially abundant proteins (DAPs) were observed in the control and aged groups, respectively. Interestingly, a higher activity in metabolic processes, protein synthesis, transcription, cell growth/division, and signal transduction were already found in imbibed embryos of control ST seeds. After AA, 132 and 64 DAPs were accumulated in imbibed embryos of both aged ST and SS seeds, respectively, which were mainly associated with cell defense, rescue, and metabolism. Moreover, 78 DAPs of ST appeared before CN and were mainly enriched in biological pathways related to the maintenance of redox and carbon homeostasis and they presented a stronger protein translation ability. In contrast, in SS, only 3 DAPs appeared before CN and were enriched only in the structural constituents of the cytoskeleton. In conclusion, a longer span of plateau phase might be obtained in seeds when proteins indicate an intense stress response before CN and include the effective maintenance of cellular homeostasis, and avoidance of excess accumulation of cytotoxic compounds. Although key proteins, inherent factors and the precise regulatory mechanisms need to be further investigated, the found proteins may also have functional potential roles during long-term seed conservation.

## Introduction

The seed is an important genetic carrier of germplasm resources which form the basis for the earliest innovations and the future developments in agriculture. Most orthodox (desiccation tolerant) seeds are conserved under low temperature and low humidity conditions (Walters et al., 2005). Wheat (*Triticum aestivum* L.) is the second most important cereal after rice, and its safe conservation is vital for sustainable development in agriculture (Li et al., 2019). To date, more than 850,000 wheat germplasm resources (accessions) have been conserved at genebanks worldwide, of which 51,000 have been stored at –18°C in the National Genebank of China (unpublished). Though, storage conditions follow international standards (FAO, 2014), seeds deteriorate (Lu et al., 2005; Walters et al., 2005; Lee et al., 2019) which is accompanied by the accumulation of reactive oxygen species (ROS), disruption of cellular membranes, collapse of defense system, and oxidative damage to sugars, lipids, proteins, and nucleic acids (Rajjou et al., 2008; Waterworth et al., 2015; Yin et al., 2017; Nagel et al., 2019). During the loss of seed viability, a reverse S-shaped curve forms that comprises an initial plateau phase (PI), a rapidly decreasing phase (PII), and a slowly decreasing phase (PIII). The transition from P-I to P-II, especially when seed viability falls below 85% of initial germination is defined as the critical node (CN) which is an important threshold for regeneration decision in genebank management (FAO, 2014; Yin et al., 2016, 2017). The wide variation in falling below CN between and within species is based on the difference of seed longevity and affects seed storability (Nagel et al., 2009; Niedzielski et al., 2009; Lee et al., 2019).

Seed storability is a complex quantitative trait and defined as the ability of seeds to maintain alive during storage. It is affected by genetic and environmental factors during plant growth, seed maturation and post-harvest, and is reflected by the variations in the physiological and biochemical status and intrinsic genetic factors of seeds, such as the testa morphology, carbohydrates, lipids, and proteins (Clerkx et al., 2004; Nagel et al., 2009; Nguyen et al., 2015; He et al., 2016; Li et al., 2017). Genetic studies revealed quantitative trait loci (QTLs) for seed storability in bread but also durum wheat being distributed on many chromosomes indicating the complexity of the trait (Landjeva et al., 2010; Rehman Arif et al., 2012, 2017; Börner et al., 2018; Zuo et al., 2018; Rehman Arif and Börner, 2019, 2020). Recently, key proteins involved in the lipid peroxidation, energy metabolism, maintenance of redox homeostasis, scavenging of cytotoxic compounds, and repair of oxidative damage have been found to play vital role in the regulation of seed storability, and have been reported in many species including *Arabidopsis*, rice, tobacco, and chickpea (Verma et al., 2013; Rissel et al., 2014; Nisarga et al., 2017; Yazdanpanah et al., 2019). For example, NADP-malic enzyme 1, aldehyde dehydrogenase (ALDH), poly (ADPribose) polymerase (PARP), and protein L-isoaspartyl-*O*-methyltransferase (PIMT), are positively correlated with seed storability by possibly avoiding oxidative damage, maintain the genomic integrity and function of proteins (Verma et al., 2013; Rissel et al., 2014; Nisarga et al., 2017; Yazdanpanah et al., 2019). However, how these key proteins contribute to the higher storability is still unclear. In a previous study, we obtained two wheat accessions (TRI 23248 and TRI 10230) from the IPK Gatersleben genebank. Their storability was identified by comparing seed viability after storage for 10 years under the long-term storage (–18°C) and ambient (20°C) conditions. Seeds of TRI 23248 germinated at 84% and the genotype was characterized as storage-tolerant (ST), and seeds of TRI 10230 had a germination of 14% and the genotype classified as storage-sensitive (SS) (Chen et al., 2018). Gene expression and proteomic based two-dimensional polyacrylamide gel electrophoresis analysis of both genotypes showed that ST seeds imbibed for 24 h had higher abundance in some proteins related with antioxidant defense system, protein destination and storage, and others, leading to extended longevity during conservation (Chen et al., 2018). Although Yin et al. (2017) showed the abundance of proteins and carbonylation patterns at and around the CN for rice, the proteomic profiles and the potential regulatory mechanisms at the CN among are still ambiguous in wheat germplasms with apparent differences in seed storability. Therefore, the aim of the study is to elucidate molecular regulations around CN, an important threshold for genebank regeneration, and to identify key proteins relevant for long storability and possible prediction tools. Therefore, we regenerated the two wheat accessions TRI 23248 and TRI 10230 and applied artificial aging (AA) treatment to gain a seed germination around CN, at 90, 80, and 60%. Imbibed embryos were extracted from AA seeds and investigated using an iTRAQ-based quantitative proteomic technology to further explain the variation in protein expression regulation before CN.

## Materials and Methods

### Plant Materials and Treatment

Two wheat accessions TRI 23248^1^ and TRI 10230^2^ obtained from the genebank of the Leibniz Institute of Plant Genetics and Crop Plant Research (IPK), Germany, were used. TRI 23248 (longer seed longevity), was termed the storage-tolerant genotype, while TRI 10230 (shorter seed longevity), was termed as the storage-sensitive genotype. Both accessions were regenerated in 2015. After harvest, the mature seeds were cleaned, dried to the same water content (7.2%) and stored at –20°C in aluminum foil bag until usage. AA treatment was performed by placing seeds in tightly closed desiccators with saturated sodium chloride solution at the bottom to obtain 75% relative humidity at 40°C for 36 days (Yin et al., 2016). Every 4 days, seeds were withdrawn from the treatment and immediately germinated to characterize sigmoidal shaped aging curve for each genotype.

### Seed Germination Test

Seed germination was performed according to the protocol of the International Seeds Testing Association (2015). Triplicate with 50 seeds were germinated at 25°C in the dark. The germination percentage (GP) of the control group was 99%. Based on GP, the viability curve of the seed was plotted to select appropriate nodes at P-I and P-II. The survival curve was fitted using Avrami equation [ln(*N0*/*N*) = (*t*/Φ)*^*n*^*, *N0* represented the average initial germination percentage, *N* was the average germination percentage for the time (*t*) in days, Φ and *n* were respectively the coefficient and exponential factor of Avrami equation] with OriginPro software (Walters et al., 2005), and then the calculated time for seed germination to decrease to 50% (P50) was obtained to illustrate the different declines between the two wheat accessions. Finally, seeds belonging to three aging groups (90, 80, and 60%) and two untreated control groups were imbibed in water at 25°C for 24 h. Subsequently, the seed embryos were extracted to further analyze the proteomic differences between the two wheat accessions.

### Protein Preparation

Total protein was extracted as described by Chen et al. (2018). Briefly, 50 embryos were ground into a fine powder in liquid nitrogen, and suspended in 50 mM Tris-HCl buffer (pH 7.5) containing 0.8 M sucrose, 5 mM EDTA, and 65 mM dithiothreitol. The suspension was mixed with tris-saturated phenol and incubated in an ice bath for 30 min. After centrifugation at 10,000 *g* for 10 min, ammonium acetate/methanol (0.1 M) was used to precipitate the protein at –20°C. Finally, the protein pellet was washed thrice with chilled acetone, and vacuum-dried. Three biological replicates were used separately. The protein pellet was dissolved in hydration buffer containing 7 M urea, 2 M thiourea, 2% CHAPS, and 1% dithiothreitol. Subsequently, it was ultrasonicated for 15 s, the protein concentration was detected by the Bradford method (Bradford, 1976).

### Protein Digestion

Proteins were digested using the filter-aided sample preparation method, as previously described by Wiśniewski et al. (2009). Briefly, the total protein samples (200 μg) diluted in hydration buffer were mixed with 50 mM Tris (2-carboxyethyl) phosphine (1:50, v/v) and incubated at 37°C for 2 h. Subsequently, 200 mM methyl methanethiosulfonate (1:100, v/v) in isopropanol was added to the mixture and incubated at room temperature in the dark for 30 min. The samples were transferred onto a 10 kDa filter (Sartorious, Göttingen, Germany) for ultrafiltration and centrifuged at 10,000 × *g* for 10 min, at 4°C. Subsequently, three wash steps were performed with 400 μL of ddH_2_O. After each wash step, centrifugation was performed at 10,000 × *g* for 20 min. Finally, 85 μL of dissolution buffer (500 mM triethylammonium bicarbonate, pH 8.5) and 2 μg of trypsin (Applied Biosystems, Foster City, CA, United States) were added to each filter. The samples were incubated at 37°C overnight for 16∼18 h. The resulting peptides were collected as the filtrate and freeze-dried into a powder for protein labeling.

### Protein Labeling

The peptide mixture was labeled with the iTRAQ reagent 8-plex multiplex kit (Applied Biosystems, United States), according to the manufacturer’s instructions. In TRI 23248 and TRI 10230, the control and aging groups were separately labeled as (ST-99%)-113, (ST-90%)-114, (ST-80%)-115, (ST-60%)-116, (SS-99%)-117, (SS-90%)-118, (SS-80%)-119, and (SS-60%)-121. The labeled samples were incubated at room temperature for 2 h and the reaction was terminated by adding 100 μL of ddH_2_O. The peptide mixtures were pooled, vacuum-frozen, and dried into powder. Protein labeling was performed in triplicate.

### Peptide Separation Using SCX Chromatography

The dried peptide mixture was reconstituted and acidified with buffer A (10 mM KH_2_PO_4_ in 25% acetonitrile, pH 3.0) and loaded onto a 4.6 × 100 mm polysulfethyl column (5 μm, 200 Å, PolyLC, Inc., Columbia, MD, United States). The peptides were eluted at a flow rate of 1 mL min^–1^ with a gradient of 0∼5% buffer B (2 M KCl and 10 mM KH_2_PO_4_ in 25% ACN, pH 3.0) for 1 min, 5∼30% buffer B for 20 min, 30∼50% buffer B for 5 min, 50% buffer B for 5 min, 50∼100% buffer B for 5 min, and 100% buffer B for 10 min. The elution was monitored by measuring the absorbance at 214 nm of fractions collected every 1 min. Thirty-two fractions were finally combined into 16 pools and desalted on C18 Cartridges [Empore SPE Cartridges C18 (standard density), bed I.D. 7 mm, volume 3 mL, Sigma, St. Louis, MO, United States]. All fractions were dried and stored at –80°C until further analysis.

### NanoRPLC-MS/MS Analysis

NanoRPLC-MS/MS experiments were performed on a Triple-TOF 5600 + system (AB SCIEX, Framingham, MA, United States) coupled to an Eksigent nanoLCUltra binary pump system (AB SCIEX). The peptide mixture was loaded onto a trap column (Eksigent, 100 × 20 mm) connected to an analytical column (Eksigent, 75 μm × 150 mm), in mobile phase A (2% acetonitrile, 0.1% formic acid), at a flow rate of 2 μL min^–1^. Both the trap and analytical columns were filled with MAGIC C18 – AQ 5 μm 200 Å phase (MICHROM Bioresources, Inc., Auburn, CA, United States). The peptides were separated with mobile phase B (98% acetonitrile, 0.1% formic acid) at a linear gradient of 5 – 80%, for 90 min at a flow rate of 300 nL.min^–1^, which was controlled by the Intelli Flow technology. The MS analysis was performed in the information-dependent mode. Mass spectrometry (MS) spectra were acquired across the mass range of 350∼1500 m/z in high-resolution mode (>30,000) using 250 ms as the accumulation time per spectrum. A maximum of 40 precursors per cycles was chosen for fragmentation from each MS spectrum, with a minimum accumulation time of 50 ms for each precursor, and dynamic exclusion for 20 s. Tandem mass spectra were recorded in high sensitivity mode (resolution > 15,000) with rolling collision energy. Data are available via ProteomeXchange with identifier PXD025860.

### Protein Identification

The peptides were identified using ProteinPilot 4.5 software (AB SCIEX) with the Paragon database search algorithm and an integrated false discovery rate (FDR) analysis. Only unique peptide sequences were used as evidence for protein identification. The data were run against the UniProt database. The user defined parameters as follows: (i) sample type, iTRAQ 8-plex (peptide labeled); (ii) cysteine alkylation, MMTS; (iii) digestion, trypsin; (iv) instrument, Triple-TOFTM 5600; (v) special factors, none; (vi) species, none; (vii) ID focus, biological modifications; (viii) database, Uniprot-*Triticum aestivum* 20170920.fasta; (ix) search effort, thorough; and (x) detected protein threshold, Unused ProtScore (conf) > 0.05 (10%). For determination of the false discovery rate (FDR), the data were searched against concatenated databases *in silico* for decoy sequences, by on-the-fly reversal, automatically by the software. Only proteins with a 1% global FDR from fit were used for further analysis.

### Protein Quantification Analysis

Protein abundance was calculated as the sum of the peak areas of unique peptides (contribution > 0, confidence = 99%, and annotated as ‘‘auto’’), followed by logarithmic transformation (Log10), and normalization using the median protein abundance for each label, to correct for systematic errors. Proteins present in two or three biological replicates were analyzed using the Limma ranked-product approach with the online software^3^ described by Schwämmle et al. (2013). The ratio of protein abundance in the aged groups to that in the respective control groups, and between the two control groups, was used to evaluate the fold change (FC). An FDR-based estimate was used to obtain the set of significant proteins. Each protein was assigned a total of seven expression ratios with corresponding adjusted *p*-values. A moderate FC cut-off of < –1.5 was applied for lower abundance, whereas a cut off > 1.5 was applied for proteins with higher abundance. Proteins falling within these thresholds were considered as differentially abundant if the adjusted *p*-value was < 0.05 [differentially abundant proteins (DAPs)].

### Bioinformatics Analysis

The identified proteins were annotated using BLAST 2 GO software (version 5.0 basic). DAPs were grouped according to their biological function using Gene Ontology (GO) terms^4^, and mapped to reference authoritative pathways using the Kyoto Encyclopedia of Genes and Genomes (KEGG)^5^, to determine the biologically active pathways. GO enrichment analysis was performed using g: Profiler with a filtering threshold of g: SCS less than 0.05, as described by Reimand et al. (2019). Dynamic changes in DAPs were analyzed using the Short Time-series Expression Miner software and clustering algorithm by setting 20 as the maximum number of model profiles, and 0.15 as the maximum unit change in model profiles, and 0.7 as the minimum correlation in the option for clustering profiles on the OmicShare tools platform, which is a free online platform for data analysis^6^ (Ernst and Bar-Joseph, 2006).

### Statistical Analyses

High-confidence results were obtained by using moderated *t*-test (Limma) with rank products based on well-defined null hypotheses. For the aging group, two paired tests were performed within eight samples by using ST-99%-113 and SS-99%-117 as controls. For the control group, some DAPs from the intersection of the two paired tests were selected as the variation between the two materials. Differences at the level of *p* < 0.05, 0.01, and 0.001 were considered as significant, which were respectively labeled as ^∗^, ^∗∗^, and ^∗∗∗^, respectively.

## Results

### Pattern of Seed Survival Curves and Number of DAPs Differ Between ST and SS Wheat Accessions After AA Treatment

**FIGURE 1:**
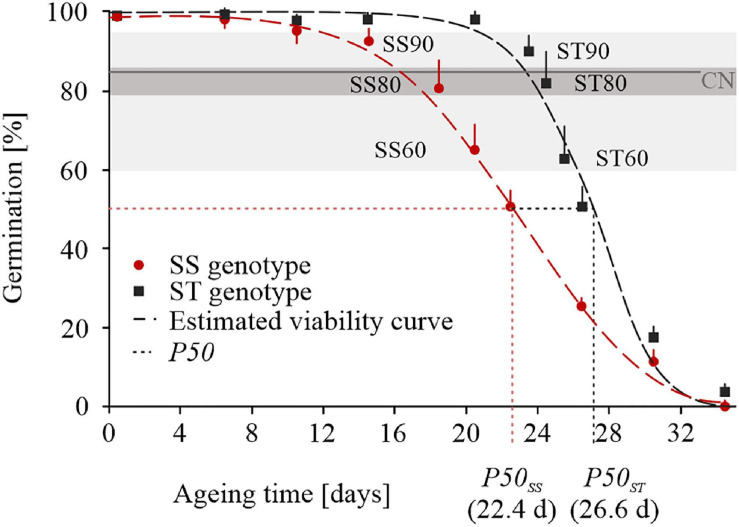
Seed viability curves and P50 of the storage-tolerant (ST) wheat accession TRI 23248 and the storage-sensitive (SS) accession TRI 10230 following artificial aging treatment. Mean and standard error of three replicates is given. ST90, ST80, ST60, SS90, SS80, and SS60 represent stages (PI, PII, PIII) around and close to the critical node (CN) of seed germination (85%) that were used to proteomic analysis. The half-viability period (P50) is given for ST and SS seeds. Color code provided in the figure.

Seed from ST and SS genotypes lost germination over an aging period of 36 days but slopes of survival curves were significantly different (Figure 1). Overall, a half-viability period (P50) was estimated to be 26.64 days and of 22.44 days for ST and SS seeds, respectively (Figure 1), confirming the previous results of seed storability after long-term cool and long-term ambient storage (Chen et al., 2018). ST seeds showed a longer plateau phase compared to SS seeds and were close to CN (85%) after 24 days (GP, 82.00% ± 8.00%). SS seeds approached CN after 18 days (GP, 80.67% ± 8.08%). In order to reveal differences in the proteome profile at the CN of ST and SS wheat accessions, imbibed embryos were extracted for comparative proteomics. Embryos were prepared from three stages around and close to CN and extracted from seeds imbibed for 24 h. For ST wheat, seeds of an untreated control were used and seeds aged for 23 days (GP, 90.00% ± 4.00% defined as ST90), for 24 days (GP, 82.00% ± 8.00% defined as ST80), for 25 days (GP, 62.67% ± 8.33% defined as ST60). For SS wheat, seeds from untreated control and seeds aged for 14 days (GP, 92.67% ± 3.06% defined as SS90), for 18 days (GP, 80.67% ± 8.08% defined as SS80), for 20 days (GP, 65.00% ± 5.00% defined as SS60) were used for embryo extraction.

**FIGURE 2:**
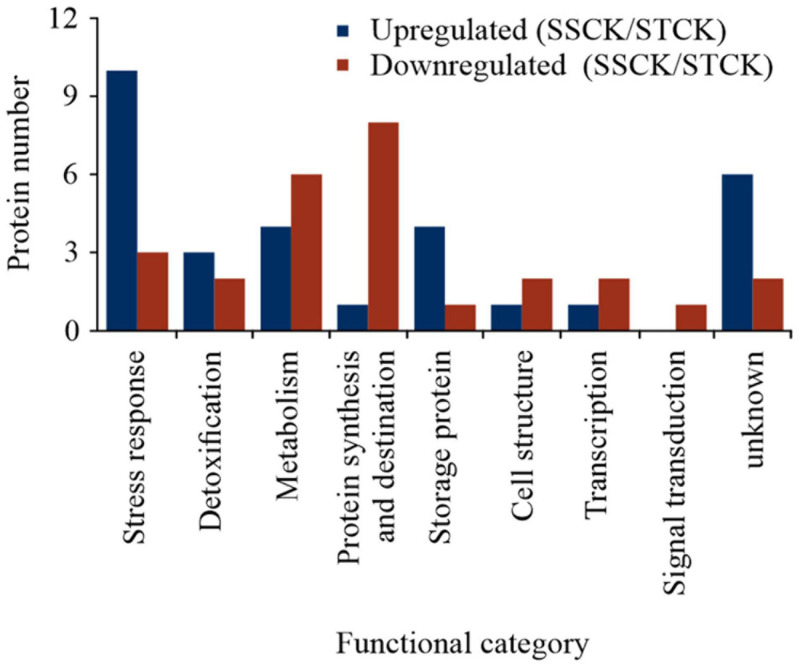
Functional classification of identified differentially abundant proteins between the untreated control group of the storage-tolerant (ST) and of the storage-sensitive (SS) embryos with upregulation (blue) or downregulation (red) according to Bevan et al. (1998) and Zhang et al. (2016). SSCK/STCK represented the comparison of protein abundance between the control group of ST and SS seeds.

**FIGURE 3:**
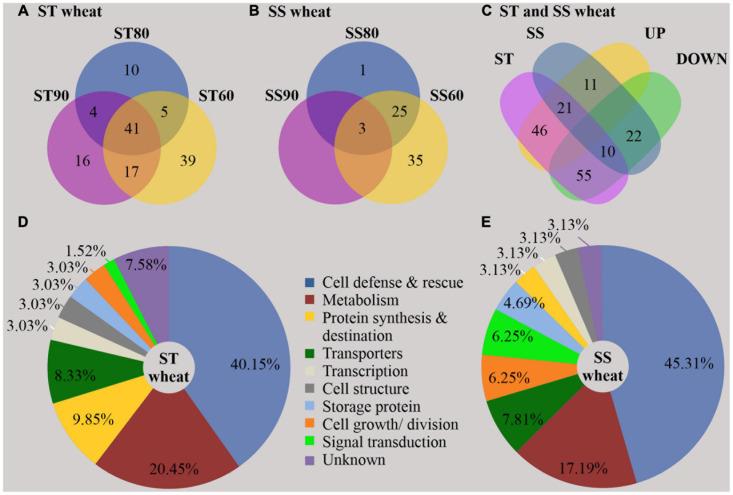
Distribution and function of differentially abundant proteins (DAPs) resulting from artificial aging treatment of storage-tolerant (ST) and storage-sensitive (SS) seed. The Venn diagram shows the distribution of DAPs in ST **(A)** and SS **(B)** embryos around (SS90/ST90 and SS60/ST60) and close to the critical node (SS80/ST80 and their intersection) **(C)**. The Pie chart shows the functional classification according to Bevan et al. (1998) and Zhang et al. (2016). 132 DAPs were found in imbibed embryos of ST seeds **(D)**, and 64 DAPs in imbibed embryos of SS seeds **(E)**. % is given of the total number of proteins in ST and SS embryos, respectively, UP, upregulated DAPs; and DOWN, downregulated DAPs.

A total of 2,118 proteins were identified after merging the data from all experimental groups (Supplementary File 1). By analyzing the embryos of imbibed SS and ST seeds, overall, 57 DAPs were identified in the untreated control groups (Figure 2 and Supplementary File 2), and 165 DAPs were obtained as result of seed aging (Figure 3 and Supplementary File 3) and are visualized in a heatmap (Supplementary Figure 1). In imbibed embryos of aged ST and SS seeds, 31 proteins were differentially accumulated in both accessions; 21 were upregulated and 10 were downregulated (Figure 3C). Besides, 101 DAPs (46 upregulated and 55 downregulated) occurred only in embryos of the aged ST seeds (Figure 3C), and 33 DAPs (11 upregulated and 22 downregulated) were found only in embryos of the aged SS seeds (Figure 3C).

In imbibed embryos of aged ST seeds, the abundance of in total 78 proteins changed when GP declined to ST90 (Figure 3A), while only 41 DAPs maintained the significant changes in all treated groups (PI to III). In addition, 10 DAPs were significant when GP approached CN at ST80, and 39 DAPs appeared only at ST60. However, in embryos of aged SS seeds (Figure 3B), only three DAPs occurred in all treated groups, 26 DAPs were presented when GP was close to CN at SS80 and 35 DAPs appeared at the SS60.

### Functional Comparison of DAPs at the CN of Seed Viability From Two Wheat Accessions Differing in Storability

**FIGURE 4:**
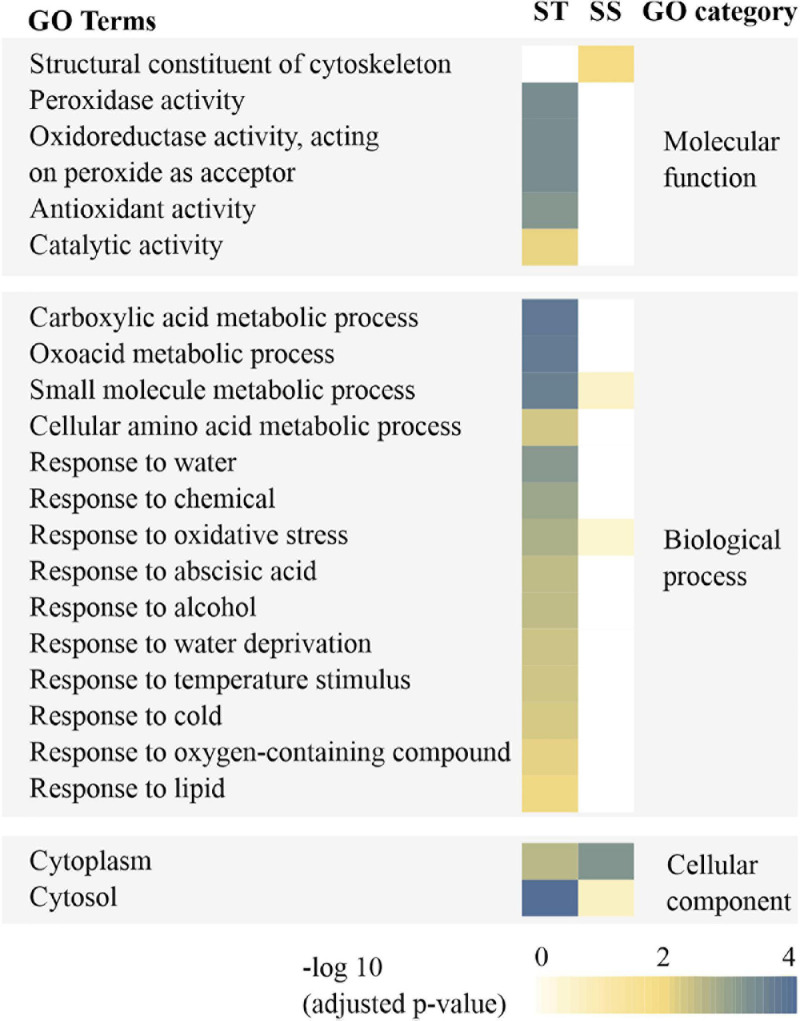
Enrichment analysis for GO annotation of differentially abundant proteins (DAPs) resulting from imbibed embryos of aged storage-tolerant (ST) and storage-sensitive (SS) wheat accessions. GO categories were assigned as significant thresholds for correlation statistics (*p* < 0.05).

Gene Ontology (GO) annotations and category were performed according to Bevan et al. (1998) and Zhang et al. (2016). By contrast, these results showed that certain proteins associated with metabolism, protein synthesis and destination, transcription, cell structure, and signal transduction were more abundant in embryos of untreated imbibed ST seeds, while embryos of untreated imbibed SS seeds had a relatively higher abundance of proteins associated with the stress response and storage proteins indicating that embryos of ST and SS had already a different proteome after harvest (Figure 2 and Supplementary File 2). After seed aging, DAPs associated with cell defense and rescue, and were most abundant and accounted for 40.15% in embryos obtained from aged ST seeds (Figure 3D) and 45.31% in embryos obtained from aged SS seeds (Figure 3E). DAPs associated for metabolism accounted for 20.45 and 17.19% in embryos obtained from aged ST and SS seeds, respectively. Interestingly, DAPs involved in protein synthesis and destination represented 9.85% in embryos from aged ST seeds; whereas, in aged SS seeds, the percentage of DAPs associated with this functionality was very low, only 3.13%. Moreover, embryos obtained from aged ST seeds were also significantly enriched in molecular functions related to antioxidant, oxidoreductase, and peroxidase activities, and mainly participated in certain biological pathways associated with stress response and metabolic processes (Figure 4). The DAPs in embryos obtained from aged SS seeds were only enriched in the structural constituents of the cytoskeleton (Figure 4). Concluding, the results indicated that imbibed embryos of ST seeds have a higher ability to perceive, defend, and repair various damages around CN, and ST seeds might be able to maintain this ability for a longer storage period compared to SS seeds.

### Dynamic Changes of DAPs at the CN of Seed Viability From Two Wheat Accessions Differing in Storability

**FIGURE 5:**
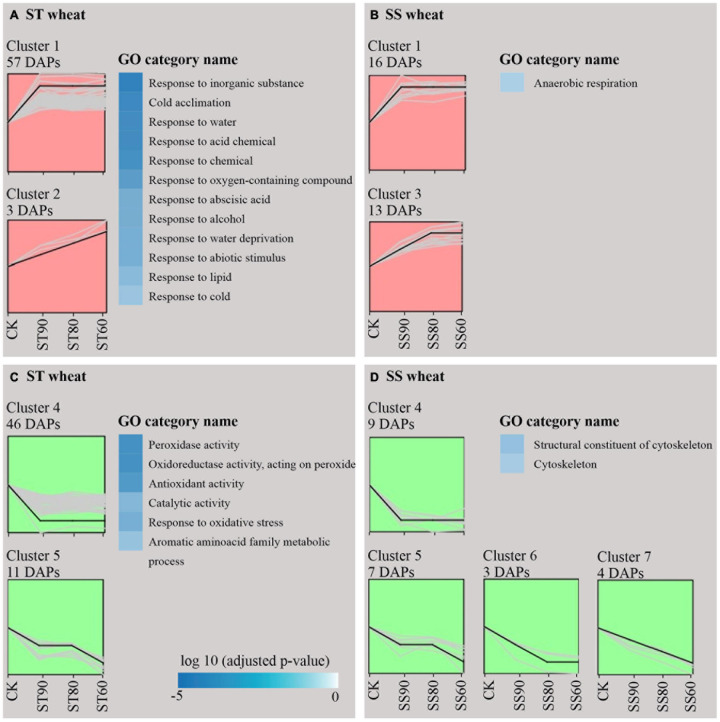
Cluster analysis and GO categories for differentially abundant proteins (DAPs) around (SS90/ST90 and SS60/ST60) and close to the critical node (SS80/ST80) extracted from embryos of imbibed seeds. Up-accumulation of aged storage-tolerant (ST) seeds **(A)** and aged storage-sensitive (SS) seeds **(B)** and down-accumulation of ST seeds **(C)** and SS seeds **(D)**. GO category represent annotation in DAPs with significant trend of upregulation (red) or downregulation (green).

To gain deeper insight into the dynamic changes in proteins between imbibed embryos of SS and ST seeds during the response to AA treatment, we clustered the DAPs result from aging based on their abundance data using the STEM clustering algorithm, and the actual size based *p*-value protein enrichment was computed by hypothesis testing. Based significant thresholds for correlation statistics (*p* < 0.05) (Ernst and Bar-Joseph, 2006), the results showed that all the DAPs from imbibed embryos of aged ST and SS seeds were clustered into 13 profile models, of which 7 trends presented significant change including 141 DAPs were obtained (Figure 5, Tables 1–4, and Supplementary File 4).

**TABLE 1:** Differentially abundant proteins in embryos of ST seeds with significant trend in up-regulation during artificial aging at 40°C and 75% relative humidity.

**Accession**	**Protein description**	**Function**	**Fold change**			
			**ST90/STCK**	**ST80/STCK**	**ST60/STCK**	**SSCK/STCK**
**Cell defense and rescue**						
A0A0H4MAT1	Dehydrin 1	Stress response	1.73*	1.61	1.56	2.16
Q41579	Dehydrin 4	Stress response	2.34*	1.83	2.06*	1.96
W5GW81	Dehydrin 4	Stress response	1.67*	1.44	1.75*	1.63
W5ERW2	Dehydrin 6	Stress response	1.61*	1.27	1.62*	1.36
A0A1D5SU11	Em protein	Stress response	2.51**	2.40*	2.63**	1.60
A0A1D5WW07	Late embryogenesis abundant protein EMB564	Stress response	5.53*	5.72*	4.99*	2.44*
W5CL83	Em-like protein	Stress response	2.47*	2.24	3.43**	1.34
A0A1D6C183	16.9 kDa class I heat shock protein 1-like	Stress response	2.26*	1.77	2.05	1.48
A0A1D6CJL6	16.9 kDa class I heat shock protein 1-like	Stress response	1.83*	1.72*	1.85**	1.40
W5BBE4	23.2 kDa heat shock protein	Stress response	2.40**	2.13**	1.62*	3.94***
A0A1D6REH6	Embryonic protein DC-8	Stress response	2.26*	1.68	2.03*	1.57
E6Y2L2	Seed maturation protein PM41	Stress response	2.74*	2.05	1.87	1.96
W5EJM2	Seed maturation protein PM41	Stress response	1.56*	1.39	1.50*	1.49*
W4ZQK5	Small hydrophilic plant seed protein	Stress response	2.16*	1.69	1.79	1.09
A0A1D5XWV5	Late embryogenesis abundant protein D34	Stress response	1.85*	2.03*	2.18**	1.51
W5FLF3	Late embryogenesis abundant protein D34	Stress response	1.49	1.27	1.59*	1.51
A0A1D5ZRK3	Late embryogenesis abundant protein 3	Stress response	1.92	1.92	2.40*	0.71
A0A1D5URY0	LEA domain-containing protein	Stress response	1.73	1.75	1.95*	1.38
A0A1D6CKF4	Oleosin 16.4 kDa-like	Stress response	1.95**	1.85**	1.49*	1.69*
A0A1D5STX4	Predicted protein	Stress response	1.83***	1.85**	2.19***	2.11***
Q6W8Q2	1-Cys peroxiredoxin PER1	Detoxification	1.53	1.63	1.61*	1.21
A0A1D5ZD36	Glutathione-dependent formaldehyde	Detoxification	1.51*	1.41	1.46	1.29
	activating enzyme					
A0A1D6CB85	Lactoylglutathione lyase	Detoxification	1.77*	1.44	1.50*	1.41
A0A1D5TZ33	Protein-L-isoaspartate *O*-methyltransferase	Cell rescue	1.54	1.56	1.96*	1.07
**Metabolism**						
W5A6D0	Aldose reductase	Carbohydrate	1.96*	2.14*	2.00*	1.47
W4ZUK2	Glucose and ribitol dehydrogenase homolog	Carbohydrate	1.36	1.44	1.72*	1,26
A0A1D5XI63	Ricin B-like lectin R40C1	Carbohydrate	2.05*	2.63**	1.72	1.58
C7C4 × 1	Glyceraldehyde-3-phosphate dehydrogenase	Energy	1.81*	1.32	1.57	1.22
Q1PBI3	Glucose-6-phosphate isomerase, cytosolic	Energy	1.69*	1.27	1.32	1.41
**Protein synthesis and destination**						
A0A1D5Z340	60S ribosomal protein L7a	Protein synthesis	2.01*	1.52	1.65	1.53
A0A1D6ASL7	60S ribosomal protein L6-like	Protein synthesis	1.26	1.55*	1.32	1.29
A0A1D5Z5S3	D-aminoacyl-tRNA deacylase	Protein synthesis	1.16	1.33	1.60*	1.25
**Transporters**						
A0A1D6CN29	Copper transport protein ATX1	Ions transport	1.68*	1.83*	1.58*	1.44
W5EC46	Non-specific lipid-transfer protein 4-like	Lipids transport	1.89*	2.08*	1.34	1.81
W5F7M9	Putative chaperone GrpE type 2	Protein transport	1.06	1.25	2.00*	1.33
**Storage protein**						
I6QQ39	Globulin-1 S allele	Nutrient reservoir	3.30*	2.07	2.8	1.11
A0A1D5ZZT8	Globulin-1 S allele-like	Nutrient reservoir	1.92*	1.85	2.20*	1.39
**Unknown**						
A0A1D5XIA7	Predicted protein	Unknown	1.56	1.61	1.89*	1.26
A0A1D6AI79	Predicted protein	Unknown	1.80**	1.41	1.97**	1.46
A0A1D6AZR7	Predicted protein	Unknown	2.43*	2.25	3.45*	2.02
W5CQP6	Predicted protein	Unknown	1.66*	1.73*	1.72*	1.61
W5BGG8	Uncharacterized protein LOC109781115	Unknown	1.68*	1.33	1.57	0.71

ST90/STCK, ST80/STCK, and ST60/STCK respectively represented the comparison of protein abundance between the aging groups with germination percentage of ∼90, ∼80, ∼60% and the control group. *, **, *** represent that the significant fold change of protein abundance was at the level of *p***<** 0.05, 0.01, 0.001, respectively.

**TABLE 2:** Differentially abundant proteins in embryos of ST seeds with significant trend in down-regulation during artificial aging at 40°C and 75% relative humidity.

**Accession**	**Protein description**	**Function**	**Fold change**			
			**ST90/STCK**	**ST80/STCK**	**ST60/STCK**	**SSCK/STCK**
**Cell defense and rescue**						
A0A1D5YB80	Heat shock 70 kDa protein, mitochondrial-like	Stress response	0.52**	0.55**	0.48***	0.47***
W5FEE5	Heat shock cognate 70 kDa protein 2-like	Stress response	0.36*	0.41*	0.48***	0.52
W5FA12	Em-like protein	Stress response	0.35*	0.40*	0.26**	0.42
W5H102	Soluble inorganic pyrophosphatase 6, chloroplastic	Stress response	0.73	0.69	0.39*	0.64
A0A1D5U1N2	GYF domain-containing protein	Stress response	0.69	0.56*	0.56*	0.79
A0A1D5V9V3	Peroxidase 2-like	Detoxification	0.45*	0.50*	0.40*	0.60
C3VQ52	Ascorbate peroxidase	Detoxification	0.59**	0.64*	0.50*	0.78
A0A1D5TUQ5	Cationic peroxidase SPC4-like	Detoxification	0.61	0.49*	0.64*	0.56
A0A1D5UU04	Cationic peroxidase SPC4-like	Detoxification	0.71	0.61*	0.49*	0.99
A0A1D6A4I6	Glutathione S-transferase zeta class	Detoxification	0.78	0.58*	0.61*	0.82
Q8GTB8	Glutathione S-transferase 3-like	Detoxification	0.40**	0.56*	0.58*	1.11
**Metabolism**						
A0A1D6C6R0	Indole-3-glycerol phosphate synthase, chloroplastic	Amino acid	0.61	0.56*	0.56*	0.85
Q8W430	Sucrose 1-fructosyltransferase	Carbohydrate	0.28*	0.26*	0.56*	0.32*
A0A1D5RYR3	UDP-glucuronic acid decarboxylase 2	Carbohydrate	0.61	0.58*	0.26*	0.65
A0A1D6CYP4	Probable galactinol–sucrose galactosyltransferase 1	Carbohydrate	0.46*	0.62	0.58*	0.69
A0A1D6RST6	Mannose-1-phosphate guanylyltransferase 1	Carbohydrate	0.64	0.49*	0.62	0.69
W5FDW8	UDP-glucose 6-dehydrogenase 4	Carbohydrate	0.3**	0.43*	0.49*	0.67
W5GW19	Predicted protein	Nucleotide	0.74	0.69	0.43*	0.72
A0A1D6S4A3	Deoxyuridine 5’-triphosphate nucleotidohydrolase	Nucleotide	0.68	0.55*	0.69	0.60
A0A1D5Z4H3	Lipoxygenase 2	Lipid	0.33**	0.41*	0.55*	0.59
A0A1D5RUI5	3-ketoacyl-CoA thiolase 2, peroxisomal	Lipid	0.35	0.32*	0.41*	0.46
A0A1D5TUT9	Anthocyanidin reductase	Secondary metabolite	0.57	0.52*	0.32*	0.72
A0A1D5UWN7	Phospho-2-dehydro-3-deoxyheptonate aldolase 2	Secondary metabolite	0.48*	0.44**	0.52*	0.56
A0A1D5Z1D2	Anthranilate synthase alpha subunit 1, chloroplastic	Secondary metabolite	0.77	0.77*	0.44**	0.94
A0A1D6RQI0	3-dehydroquinate dehydratase/shikimate dehydrogenase, chloroplastic-like isoform X1	Secondary metabolite	0.61***	0.69**	0.77*	0.57***
A0A1D5U6U1	Adenylylsulfate kinase	Secondary metabolite	0.58	0.39*	0.69**	0.65
**Protein synthesis and destination**						
A0A1D5YFC1	Phenylalanine–tRNA ligase alpha subunit, cytoplasmic	Protein synthesis	0.89	0.55*	0.69	0.91
W5AC26	Histidine–tRNA ligase, cytoplasmic	Protein synthesis	0.42*	0.67	0.55*	0.70
Q8L806	40S ribosomal protein S18	Protein synthesis	0.09***	0.12***	0.67	0.10***
A0A1D5RS63	Ribosomal L1 domain-containing protein 1-like	Protein synthesis	0.87	0.62*	0.12***	0.67
W5ATU2	Polyadenylate-binding protein RBP47	Protein synthesis	0.57*	0.43**	0.62*	0.60
A0A1D6BMF2	Protease 2	Protein degradation	0.46*	0.58	0.43**	0.73
W5EJA6	ATP-dependent Clp protease proteolytic subunit 5	Protein degradation	0.77	0.55*	0.58	0.92
W5ASV0	Oryzain alpha chain	Protein degradation	0.37**	0.43*	0.55*	0.67
**Transcription**						
A0A1D6CRD4	RNA-binding (RRM/RBD/RNP motifs) family protein	mRNA process	0.60*	0.72	0.43*	0.87
A0A1D5UZA9	Glycine-rich RNA-binding protein 4, mitochondrial-like	RNA bind	0.71	0.77	0.72	0.66
A0A1D6C5W5	RNA exonuclease 4	rRNA synthesis	0.95	0.56*	0.77	0.63
A0A1D6AYF2	KH domain-containing protein	mRNA synthesis	0.26*	0.33	0.56*	0.24*
**Transporters**						
A0A1D5YJB1	AP-2 complex subunit alpha-2-like	Protein transporter	0.58*	0.68	0.33	0.65
A0A1D5S4G8	Golgi apparatus membrane protein-like protein ECHIDNA	Others	0.66	0.47*	0.68	0.64
A0A1D5UDS7	Mitochondrial import inner membrane translocase subunit TIM44-2	Others	0.73	0.58*	0.47*	0.73
A0A1D5SL30	Mitochondrial dicarboxylate/tricarboxylate transporter DTC-like	Others	0.69	0.61*	0.58*	0.79
**Cell structure**						
A4K4Y1	Alpha tubulin1	Cytoskeleton	0.57	0.50*	0.61*	0.63
A0A1D5YN83	Annexin D5	Others	0.76	0.43*	0.50*	0.57
**Cell growth/division**						
A0A1D5ST90	DNA replication licensing factor MCM3	DNA replication	0.36**	0.40*	0.43*	0.57
W5FPI3	DNA replication licensing factor MCM7	DNA replication	0.28***	0.41**	0.40*	0.52
**Unknown**						
A0A1D5UJV6	Naked endosperm1	Unknown	0.65	0.59	0.41**	0.69

ST90/STCK, ST80/STCK, and ST60/STCK respectively represented the comparison of protein abundance between the aging groups with germination percentage of ∼90, ∼80, ∼60% and the control group. *, **, *** Represent that the significant fold change of protein abundance was at the level of *p***<** 0.05, 0.01, 0.001, respectively.

**TABLE 3:** List of common differentially abundant proteins with significant trend in both in embryos of ST and SS seeds during artificial aging at 40°C and 75% relative humidity.

**Accession**	**Protein description**	**Function**	**Fold change**						
			**ST90/STCK**	**ST80/STCK**	**ST60/STCK**	**SS90/SSCK**	**SS80/SSCK**	**SS60/SSCK**	**SSCK/STCK**
**Cell defense and rescue**									
A0A1D5Z500	Late embryogenesis abundant protein D-34	Stress response	3.27***	3.08***	4.13***	1.24	2.06	1.89*	2.47**
W5FP47	Late embryogenesis abundant protein D-34	Stress response	1.92**	1.51	1.86**	1.25	1.60*	1.63*	1.47
A0A1D6CR43	11 kDa late embryogenesis abundant protein	Stress response	1.90*	2.11**	1.99**	2.00**	2.18***	2.10***	1.22
A0A1D5SU52	Late embryogenesis abundant protein B19.4	Stress response	4.60***	3.84***	4.92***	1.43	1.71*	1.85*	1.67
W5A4Z8	Late embryogenesis abundant protein B19.4	Stress response	3.40**	2.82*	3.32*	1.39	1.84*	1.88*	1.07
W5CUW2	Late embryogenesis abundant protein 14-A	Stress response	1.17	1.48	2.03*	1.40	2.55*	2.37*	0.90
A0A1D6BIR9	Seed maturation protein	Stress response	2.74	2.92	3.57*	1.25	2.56*	2.89*	1.93
A0A1D6CUR4	Seed maturation protein	Stress response	2.37**	1.93*	2.45**	1.74	2.65***	2.99***	1.48
A0A1D6SDR0	Seed maturation protein	Stress response	1.92*	1.75	2.50**	1.79*	2.99***	3.39***	1.31
Q53WS3	Early methionine-labeled polypeptide	Stress response	6.30***	6.38***	6.08***	1.14	1.73	1.65*	2.73*
Q9ZR70	Early methionine-labeled polypeptide	Stress response	2.15*	1.87*	2.41**	1.79	2.99**	3.67***	1.86
A0A1D5RSF5	Predicted protein	Stress response	2.72**	2.30*	3.12**	1.41	1.68	1.90*	1.08
W5FZJ8	DnaJ protein homolog	Stress response	0.48*	0.49*	0.53*	0.69	0.60*	0.55*	0.71
A0A1D6BJM1	Peroxidase 16	Detoxification	0.51**	0.57*	0.51**	0.83	0.68	0.62*	0.85
W5B6C4	Peroxidase 16	Detoxification	0.59	0.70	0.39*	0.62	0.53*	0.41**	0.94
W5EJK0	L-ascorbate peroxidase 1,	Detoxification	0.27**	0.31**	0.28**	0.56	0.41**	0.39***	0.53*
**Metabolism**									
A0A077RWS5	*S*-adenosylmethionine synthase 1	Amino acid	0.40**	0.44*	0.37**	0.74	0.66*	0.57*	0.57
W5AFS8	Aldose reductase	Carbohydrate	2.36**	2.30**	2.17**	2.14	2.24*	1.79*	1.49
A0A1D6BAP2	Alpha-amylase 1	Carbohydrate	0.22*	0.41	0.32	0.43	0.77	0.22*	0.50
W5FEY4	Predicted protein	Carbohydrate	0.27**	0.35**	0.30**	0.72	0.53	0.36*	0.59
A0A077RVB3	UDP-arabinopyranose mutase 1-like	Carbohydrate	0.60	0.66	0.46*	0.53	0.61*	0.78	0.72
**Cell growth/division**									
A0A1D5UQF4	Topless-related protein 2	Cell growth	4.47**	4.10**	3.70*	1.73	2.13*	2.20*	2.01
A0A1D5U093	Predicted protein	Cell growth	3.60*	3.11	3.20*	1.43	2.28*	2.25*	1.63
**Signal transduction**									
A0A1D5U8D4	Probable protein phosphatase 2C 58	Phosphatases	2.22	2.07	2.75*	1.22	1.94*	2.12*	1.44
**Storage protein**									
A0A1D5YEH0	Globulin-1 S allele	Nutrient reservoir	2.66**	2.23*	2.36**	1.26	1.49	1.72*	1.79
**Transporters**									
A0A1D6CVG5	ABC transporter F family member 1	ABC-type	0.47***	0.60**	0.50***	0.66*	0.60***	0.56***	0.86
**Cell structure**									
A0A1D5WND0	Tubulin beta chain	Cytoskeleton	0.29**	0.38**	0.49	0.57	0.46**	0.48**	0.65
**Unknown**									
A0A1D6BIB2	Hypothetical protein TRIUR3_06375	Unknown	2.59***	2.32**	2.19**	1.52	2.56***	2.49***	1.58

ST90/STCK, ST80/STCK, ST60/STCK, SS90/SSCK, SS80/SSCK and SS60/SSCK, respectively, represented the comparison of protein abundance between the aging groups with germination percentage of ∼90, ∼80, ∼60% and the control group in ST and SS seeds. *, **, *** Represent that the fold change of protein abundance was at the level of *p***<** 0.05, 0.01, 0.001, respectively.

**TABLE 4:** List of SS- specific differentially abundant proteins with significant trend in up- and down-regulation during artificial aging at 40°C and 75% relative humidity.

**Accession**	**Protein description**	**Function**	**Fold change**			
			**SS90/SSCK**	**SS80/SSCK**	**SS60/SSCK**	**SSCK/STCK**
**Cell defense and rescue**						
A0A1D5YGW1	Late embryogenesis abundant protein D-34	Stress response	1.55	1.94*	2.00*	1.43
A0A1D5YW17	Late embryogenesis abundant protein D-34	Stress response	1.49	1.85*	1.87*	1.21
A0A1D6RWC3	Late embryogenesis abundant protein D-34	Stress response	1.47	1.84*	2.38**	1.53
A0A1D5XF71	Late embryogenesis abundant protein D-34	Stress response	1.35	1.72	1.91*	1.24
A0A080YUM7	Calmodulin-binding protein 60 F-like isoform X2	Stress response	2.14	2.04	2.2*	0.53
**Metabolism**						
A0A1D5WYC0	Phosphoglucomutase, cytoplasmic	Carbohydrate	1.88	1.6	1.82*	0.83
W5E174	Inositol-3-phosphate synthase	Carbohydrate	2.68	1.89	2.03*	0.75
**Storage protein**						
W5EST8	Globulin-1 S allele	Nutrient reservoir	1.88	1.61*	1.82*	1.21
B7U6L5	Globulin-1 S allele	Nutrient reservoir	1.89	1.66	1.76*	4.13***
**Signal transduction**						
A0A1D5ZUF9	Ribosome-binding ATPase YchF	Mediators	1.58	1.6	1.78*	0.50
**Transporters**						
W5EMC7	Outer envelope pore protein 16-2, chloroplastic	Others	1.36	1.3	1.53*	1.15
**Cell defense and rescue**						
W5G9P1	DnaJ protein homolog 2-like isoform X2	Stress response	0.63	0.6*	0.48**	0.97
A0A1D5YRV8	Heat shock cognate 70 kDa protein 2-like	Stress response	0.66	0.62	0.43*	0.89
W5FIX9	Macrophage migration inhibitory factor homolog isoform X2	Stress response	0.73	0.77	0.59*	0.74
**Metabolism**						
A0A1D5XUF5	Outer envelope protein 64, chloroplastic	Amino acid	0.82	0.87	0.52*	1.20
A0A1D5U5C8	Probable pyridoxal 5′-phosphate synthase subunit PDX1.1	Secondary metabolite	0.74	0.67*	0.59*	0.67
**Transporters**						
Q9ZR95	Aquaporin TIP1-1	Ions transport	0.74	0.66	0.61*	1.26
W5GYF2	ADP, ATP carrier protein, mitochondrial	Others	0.61	0.63	0.59*	1.12
**Signal transduction**						
A0A1D6BU25	Profilin	Mediators	0.93	0.89	0.64*	0.98
A0A1D5SCB2	DNA-binding protein DDB_G0278111	Mediators	0.83	0.69	0.49*	0.97
**Protein synthesis and destination**						
Q7XYD5	60S acidic ribosomal protein P2B	Protein synthesis	0.88	0.65	0.58*	1.33
**Transcription**						
W5CRM8	Glycine-rich RNA-binding protein 4, mitochondrial	mRNA process	0.80	0.88	0.58*	0.91

SS90/SSCK, SS80/SSCK, and SS60/SSCK, respectively, represented the comparison of protein abundance between the aging groups with germination percentage of ∼90, ∼80, ∼60% and the control group. *, **, *** Represent that the significant fold change of protein abundance was significant respectively at the level of *p***<** 0.05, 0.01, 0.001, respectively.

Comparison between the two accessions showed that common 28 DAPs were significantly overrepresented in the profiles with apparent up-regulated or down-regulated changes (Table 3). Interestingly, the majority of ST DAPs including 10 late embryogenesis abundant (LEA) proteins, aldose reductase, topless-related protein 2 and others were enriched in cluster 1 that displayed an increase at ST90 and maintained the level of abundance close to CN at ST80 and ST60. One LEA protein was enriched in cluster 2 that displayed a continuous increase during germination drop of ST seeds (Figure 5A). However, for embryos of aged SS seeds, 14 DAPs including 8 LEA proteins, 1 Probable protein phosphatase 2C 58, 1 Globulin-1 S allele and others had the same up-regulated profile (cluster 1), only 2 LEA proteins 1 aldose reductase and 1 topless-related protein 2 was enriched in cluster 3 that displayed an continuous increase at SS90 and close to CN at SS80, then maintained the level of abundance at SS60 (Figure 5B). Besides, 2 peroxidase-related proteins and 2 carbohydrate metabolism-related proteins were down-regulated at ST90, and then maintained the level of abundance close to CN at ST80 and ST60 in embryos of aged ST seeds (Figure 5C, cluster 4), but they displayed continuous a decrease in embryos of aged SS seeds (Figure 5D, cluster 5, 6, and 7). These proteins were mainly related to cell defense and rescue, carbohydrate metabolism and cell growth. On the whole, the change in DAPs clearly lagged in the SS accession compared to that in the ST accession (Table 3).

Comparison between the two accessions also showed that clear differences were displayed in the groups of DAPs that were specifically change in either embryos of ST or SS seeds. ST DAPs with significant up-regulation were significantly overrepresented in the cluster 1 and cluster 2 that were primarily assigned to “response to abiotic stimulus,” of which 22 DAPs related to stress response and detoxification including dehydrin (FC, 1.61∼2.34), Em protein (FC, 2.40∼5.72), small heat shock proteins (sHSPs; FC, 1.62∼2.40), 1-Cys peroxiredoxin (PER1; FC, 1.61), lactoylglutathione lyase (FC, 1.50∼1.77), and protein-L-isoaspartate *O*-methyltransferase (PIMT; FC, 1.96), and 9 DAPs associated with energy and carbohydrate metabolism, protein synthesis, and transportation, such as 60S ribosomal protein (FC, 1.55∼2.01), glyceraldehyde-3-phosphate dehydrogenase (FC, 1.81), aldose reductase (FC, 1.96∼2.14), and non-specific lipid-transfer protein 4-like (FC, 1.89∼2.08) were enriched in cluster 1 (Figure 5A and Table 1). However, for aged SS seeds, only 2 LEA proteins were significantly overrepresented in the cluster 1, and only 9 DAPs related with stress response, carbohydrate metabolism and storage proteins, such as phosphoglucomutase and inositol-3-phosphate synthase, were enriched in cluster 3, which were primarily assigned to “anaerobic respiration” (Figure 5B and Table 4). These proteins may play an important role in maintaining higher germination after storage. In addition to that, for the groups of DAPs with significant down-regulation that were specifically change in ST or SS, 38 DAPs (38/47, 80.85%) of ST were mainly enriched in cluster 4 that displayed a decrease at ST90, and only nine were enriched in cluster 5, which were primarily assigned to “antioxidant activity, response to oxidative stress, and aromatic amino acid metabolic process” (Figure 5C and Table 2); 13 DAPs of SS were enriched in cluster 4∼7, which were primarily assigned to “cytoskeleton” (Figure 5D and Table 4). The downregulated proteins mainly associated with metabolism, indicating that the inhibition of metabolic activity of imbibed embryos was not only a consequence of seed aging, but might be a strategy of avoiding rapid energy consumption for stored and *de novo* synthesis of substances to maintain germination ability after storage. These results indicate that seed aging results in a decreased the ability of maintain the normal abundance of proteins associated with antioxidant defense, whereas the response above or close to CN might endow seeds with other effective defensive mechanisms, that are more favorable for avoiding the rapid decline in seed viability and germination. Therefore, effective expression and regulation of proteins before CN plays an important role in maintaining the germination ability of ST seeds.

## Discussion

During seed conservation, changes in seed viability vary greatly among intraspecific germplasms (Nagel et al., 2009; Niedzielski et al., 2009; Lee et al., 2019). The safe conservation of germplasm resources is determined by the decline in seed viability from the original level to the CN. In a first study, to understand molecular changes during genebank storage, we had selected two wheat accessions differing in seed storability and investigated the proteome after long-term ambient and cold storage (Chen et al., 2018). In the present study, we aimed to elucidate the variations in the protein profiles around CN of seed viability by applying AA to the two regenerated wheat accessions. Overall, the variation found between the two accessions was mainly exhibited in the stress response, scavenging of cytotoxic compounds, and maintenance of redox and carbon homeostasis by multiple patterns of protein abundance.

### Seeds With Longer Plateau Phase Show Stronger Stress Response Around CN

The interaction between non-reducing sugars and certain proteins, such as LEA proteins and small heat shock proteins sHSPs influence seed longevity (Chatelain et al., 2012; Kijak and Ratajczak, 2020). LEAs are hydrophilic and heat-soluble proteins that typically accumulate at high levels during seed maturation, and equip the seeds with desiccation tolerance (Kijak and Ratajczak, 2020). In the present study, we identified 25 LEA proteins showing significantly different expression patterns in imbibed embryos of aged seeds among two accessions differing in storability. Of these, dehydrins (Pfam 00257, DHN1, DHN4, and DHN6), group II LEA proteins were specifically upregulated in ST seeds. The protective activity of dehydrin is known to be related to a largely disordered structural state with conserved, short sequence motifs (K- and H-segments), which enable to enhance the stress tolerance of plants (Andersson et al., 2020; Li et al., 2020; Murvai et al., 2020; Nguyen et al., 2020). The downregulation of seed-expressed DHN can drastically shorten seed longevity in *A. thaliana* (Hundertmark et al., 2011) supporting a key role of some dehydrins for seed storability. Thereby, dehydrins may reduce the oxidative damage which accompanies seed aging by protecting DNA, lipids, and proteomes effectively activating redox enzymes and by facilitating autophagic degradation of cargo proteins (e.g., aquaporin). Thus, we speculate that ST seeds might accumulate abundant dehydrins at the maturation stage, so as to effectively protect from various damages during seed aging and imbibition, and enabling higher germination and consequently a longer plateau phase.

Other LEA proteins, such as the embryonic protein DC-8 and seed maturation protein PM41, attributed to group III (LEA_4, Pfam 02987), were significantly upregulated in long-term stored ST seeds (Liu et al., 2019). During seed aging, these proteins may protect against the inactivation of biomacromolecules by conformational changes to enable survival (Yu et al., 2019). The Em proteins, assigned to LEA_5 (group I, pfam 00477), had a very high correlation (*r*^2^ = 0.95) with the acquisition of seed longevity (Chatelain et al., 2012) and showed also significant upregulation in aged ST seeds, enabling an effectively response to adverse environments (Liu et al., 2019). Thus, certain LEA proteins are essential for higher seed storability. However, to determine how these proteins contribute to maintaining the plateau phase of seeds, their precise regulatory networks need to be verified.

Beside LEA proteins, heat-stable polypeptides, such as sHSPs, may prevent protein aggregation during biotic and abiotic environmental stress (Waters, 2013) and correlated with the establishment of seed longevity (Chatelain et al., 2012). sHSPs with molecular weights ranging from 12 to 42 kDa function as ATP-independent molecular chaperones by methionine-rich amphipathic α-crystalline C-terminal domains (Waters, 2013). Here, we found that three sHSPs (two 16.9 kDa class I HSP1-like and one 23.2 kDa HSP) were upregulated in imbibed embryos of aged ST seeds, which may protect the seeds against ROS-mediated damage and maintain the germination and plateau phase. Indeed, during AA, the function as a chaperone at high temperature might be also considered and may to prevent irreversible thermal inactivation of some enzymes. In conclusion, these results imply that the higher abundance of LEA and sHSP determine the longer span of plateau phase in ST seed, thus, they may prolong span of seed longevity during AA and long-term conservation.

### The Maintenance of Redox Homeostasis Is a Key Aspect of Longer Plateau Phase

Excess accumulation of ROS leads to an inactivation of antioxidant defense systems and an imbalance of redox homeostasis and attack on cellular components or compartments, causing inactivation of antioxidant defense systems and ultimately aggravating the loss of seed viability (Hu et al., 2012; Xin et al., 2014; Yin et al., 2014, 2017). Downregulation of some peroxidase-related proteins damages the antioxidant system and disturbs redox homeostasis at an earlier stage, hence, effecting seed storability (Xin et al., 2014; Yin et al., 2014, 2017). In the present study, the abundance of 1-Cys peroxiredoxin (1-Cys Prx, also named PER1) was dramatically upregulated in imbibed embryos of aged ST seeds when GP declined below the CN. PER1 is considered as a seed-specific antioxidant with an important role during early water imbibition (Chen et al., 2020) and improves the resistance to stress from various environments (Chen et al., 2016). It detoxifies various ROS including H_2_O_2_, hydroxyl radicals, and alkyl hydroperoxides by cysteine residues (Chen et al., 2016). In general in wheat, the regeneration and antioxidant activity of PER1 is maintained by the reducing power of the NADPH-dependent thioredoxin reductase/thioredoxin system (Pulido et al., 2009). The expression of thioredoxin domain-containing protein was dramatically downregulated in aging SS seeds below the CN, whereas this expression remained unchanged in ST seeds. These results indicate that the ability to eliminate accumulated ROS is effectively maintained in ST seeds, although seed viability decreases to the below the CN. This ability was completely lost in SS seeds.

In organisms, various spontaneous age-related covalent modifications in proteins often cause fatal damage to their structure and function. Among the modification is the formation of abnormal isoaspartyl (isoAsp) residues, which is mainly caused by either isomerization of aspartyl residues or deamidation of asparaginyl residues (Ghosh et al., 2020). Many studies have shown that massive accumulation of isoAsp in combination with reduced PIMT activity occurs in naturally and artificially aged seeds, leading to a decrease in their germination (Verma et al., 2013). IsoAsp residues negatively affect the catalytic efficiency of antioxidant enzymes, particularly catalase and superoxide dismutase, and that of the PIMT-mediated protein repair system (Petla et al., 2016; Ghosh et al., 2020). PIMT repairs damages by restoring abnormal isoAsp residues to their normal aspartyl residues through an *S*-adenosyl-L-methionine-dependent methyl esterification reaction (Ghosh et al., 2020). The overexpression of PIMT in rice, chickpea, and *Arabidopsis* seeds contributed to improved seed traits, such as longevity and vigor (Ogé et al., 2008; Verma et al., 2013; Wei et al., 2015; Petla et al., 2016). In agreement with this, in our study, PIMT was significantly upregulated in imbibed embryos of aged ST seeds with GP below the CN. On the basis of these results, we speculated that PIMT might be a major endogenous factor to repair the damage, thereby restricting ROS accumulation and lipid peroxidation and protecting the antioxidant system in ST seeds.

In conclusion, higher abundance of proteins related to defense and repair systems in imbibed embryos of ST seeds could provide stronger potential in maintaining redox homeostasis to avoid over-accumulation of various damage, which endowing longer span of plateau phase for seeds with higher storability during conservation.

### Maintaining the Carbon Homeostasis Might Be Essential for Longer Plateau Phase During AA

In the present study, sharp upregulation in glyceraldehyde-3-phosphate dehydrogenase involved in the transfer of glyceraldehyde-3-phosphate to 1, 3-bisphosphoglycerate, was only abundant above the CN in embryos of aged ST seeds (Table 1). Cytoplasmic glyceraldehyde-3-phosphate dehydrogenase can be induced under many stress conditions, such as anoxia and salt toxicity, and is involved in seed aging (Zhang et al., 2020). Cytoplasmic glucose-6-phosphate isomerase was also upregulated significantly only above the CN in embryos of aged ST seeds (Table 1). This enzyme catalyzes the inter-conversion of glycolysis intermediates (fructose-6-phosphate, Fru-6-P) and oxidative pentose phosphate pathway intermediates (glucose-6-phosphate, Glc-6-P) (Kunz et al., 2014). Cytoplasmic phosphoglucomutase was upregulated only below CN of imbibed embryos of aged SS seeds. Phosphoglucomutase catalyzes the interconversion of Glc-6-P to Glc-1-P, which is mainly used in biosynthetic pathways (e.g., cell wall polysaccharide synthesis) together with Fru-6-P and Glc-6-P, which constitute the hexose-phosphate pool and represent an important node in primary metabolism (Kunz et al., 2014). Interestingly, this enzyme was significantly upregulated below the CN of viability in SS seeds. These results indicate that ST seeds have a strong ability to maintain highly efficient energy metabolism to assure sufficient energy resources for germination.

Many important enzymes correlated with formation of cell walls were significantly downregulated during aging. For example, below CN, common DAPs in both, in imbibed embryos from aged ST and SS seeds, were linked to the UDP-arabinopyranose mutase 1-like enzyme, important for cell wall establishment because it converts UDP-arabinopyranose to UDP-arabinofuranose (Saqib et al., 2019). More DAPs participating in the formation of arabinoxylans were downregulated in imbibed embryos from aged ST seeds. These include UDP-glucose 6-dehydrogenase 4 and UDP-glucuronic acid decarboxylase 2 that are involved in the synthesis of fructans and *myo*-inositol (Suzuki et al., 2004; Diedhiou et al., 2012). Subsequently, when GP was close to CN, mannose-1-phosphate guanylyltransferase 1 catalyzing the production of GDP-mannose, which is linked with certain secondary changes such as the cell wall composition, *N*-glycosylation, and ascorbic acid production, was also downregulated only in imbibed embryos of age ST seeds (Maeda and Dudareva, 2012). This implies that the establishment of the cell wall is seriously affected in embryos of aged SS seeds but also ST seeds. However, we assume that the difference in carbon fluxes at the CN among embryos of ST and SS seeds caused the obvious divergence in the plateau phase of the viability curve by regulating carbohydrate metabolism to maintain carbon homeostasis.

### Scavenge Cytotoxic Compounds May Cause Differences in Plateau Phase and Storability

Lipid oxidation (Wiebach et al., 2020) and peroxidation (Colville et al., 2012) are the major factor responsible for the loss of seed vigor. In our study, the downregulation of lipoxygenases, an important enzyme associated with the oxidation of polyunsaturated fatty acids, was observed only in imbibed embryos of aged ST seeds. This is consistent with the findings of previous studies in which higher suppression of lipoxygenase expression enhanced the viability and longevity (Song et al., 2007; Xu et al., 2015). In the early stage of seed aging, oxidative damage results in the massive accumulation of lipid peroxide-derived aldehydes and ketones, especially α, β-unsaturated carbonyls, which generate advanced glycation end products and advanced lipoxidation end products by aggregating with proteins and lipids (Nisarga et al., 2017; Fu et al., 2018). Furthermore, at a level below the CN, the 3-ketoacyl-CoA thiolase 2 (KAT), which participates in the last step of the β-oxidation cycle, was significantly downregulated in imbibed embryos of aged ST seeds. A previous study showed that these cytotoxic compounds seriously affected seed germination and seedling vigor by altering metabolic pathways (Nisarga et al., 2017).

Aldose reductase is a key enzyme involved in the accumulation of sorbitol by reducing aldose (e.g., glucose and glucose-6-phosphate) in the polyol pathway to enhance stress tolerance. It also participates in the detoxification of reactive carbonyl metabolites (e.g., acrolein) (Tari et al., 2010). In our study, we found that this enzyme was significantly upregulated in both ST and SS seeds; however, in ST seeds, aldose reductase always maintained a higher abundance at the three levels of around CN. Massive accumulation of methylglyoxal has also been demonstrated in aged rice seeds (Nisarga et al., 2017). This compound is formed via important metabolic pathways, such as the non-enzymatic breakdown of triose phosphate isomers during glycolysis, auto-oxidation of ketone bodies and sugars, Maillard reaction between reducing sugars and amino acids, and lipid peroxidation (Mostofa et al., 2018). Lactoylglutathione lyase (known as glyoxalase I, GLY I, EC 4.4.1.5) is involved in methylglyoxal detoxification by converting glutathione and methylglyoxal into *S*-D-lactoylglutathione (Mostofa et al., 2018). Moreover, there is a relationship between stress response and resistance in plants and the abundance of GLY I protein. In particular, tolerant genotypes demonstrate higher expression of GLY genes, proteins, and their activities (Hasanuzzaman et al., 2014; Yan et al., 2016). Similarly, only ST seeds with higher storability were significantly upregulated in the aging group, which gave the stronger ability in scavenging cytotoxic compounds to postpone the appearance of CN and rapid loss of seed viability, thereby maintaining a higher germination viability.

Based on the above results, the multiple responses in the abundance of protein related to scavenging of cytotoxic compounds provided effective protection, to obtain the longer span of plateau phase in aged ST seeds, thus prolonging span of seed longevity during conservation.

## Conclusion

In conclusion, the present study compared the variations in the loss of seed viability and proteomic profiles of imbibed embryos of aged ST and SS seeds. The results of comparative proteomics performed around the CN showed that ST seeds with prolonged plateau phase during AA have a higher ability to prevent oxidative damage and excess accumulation of cytotoxic compounds, and to maintain redox and carbon homeostasis. Therefore, we conclude that ST seed have a higher capability to deal with the stresses occurring during long-term genebank storage. However, to identify potential inherent genetic factors and the fine regulatory mechanisms for prediction tool, further investigation is needed.

## Data Availability Statement

The original contributions presented in the study are publicly available. This data can be found here: ProteomeXchange with identifier PXD025860.

## Author Contributions

XC, GY, and XX designed the experiments and analyzed the data. XC and GY conducted the experiments. XX, JH, JL, and NL contributed materials, reagents, and analysis tools. XC, AB, MN, and GY wrote the manuscript. All authors contributed to the article and approved the submitted version.

## Conflict of Interest

The authors declare that the research was conducted in the absence of any commercial or financial relationships that could be construed as a potential conflict of interest.

## Publisher’s Note

All claims expressed in this article are solely those of the authors and do not necessarily represent those of their affiliated organizations, or those of the publisher, the editors and the reviewers. Any product that may be evaluated in this article, or claim that may be made by its manufacturer, is not guaranteed or endorsed by the publisher.

## Footnotes

1
https://doi.org/10.25642/IPK/GBIS/227191

2
https://doi.org/10.25642/IPK/GBIS/10202

3
http://computproteomics.bmb.sdu.dk/Apps/LimmaRP/

4
http://www.geneontology.org/

5
http://www.genome.jp/kegg/

6
http://www.omicshare.com/tools

## Abbreviations

AA: accelerated aging treatment
CN: critical node
DAPs: differentially abundant proteins
GP: germination percentage
GLY I: lactoylglutathione lyase
isoAsp: abnormal isoaspartyl residues
LEA: late embryogenesis abundant protein
PIMT: protein L-isoaspartyl-*O*-methyltransferase
PER1: 1-Cys peroxiredoxin
ROS: reactive oxygen species
sHSPs: small heat shock proteins
ST: storage-tolerant
SS: storage-sensitive.
