# Supplementary figures and images for: Comparative Proteomics at the Critical Node of Vigor Loss in Wheat Seeds Differing in Storability
Source: Front Plant Sci. 2021 Aug 30;12:707184. doi: 10.3389/fpls.2021.707184 (PMC8435634; doi:10.3389/fpls.2021.707184)

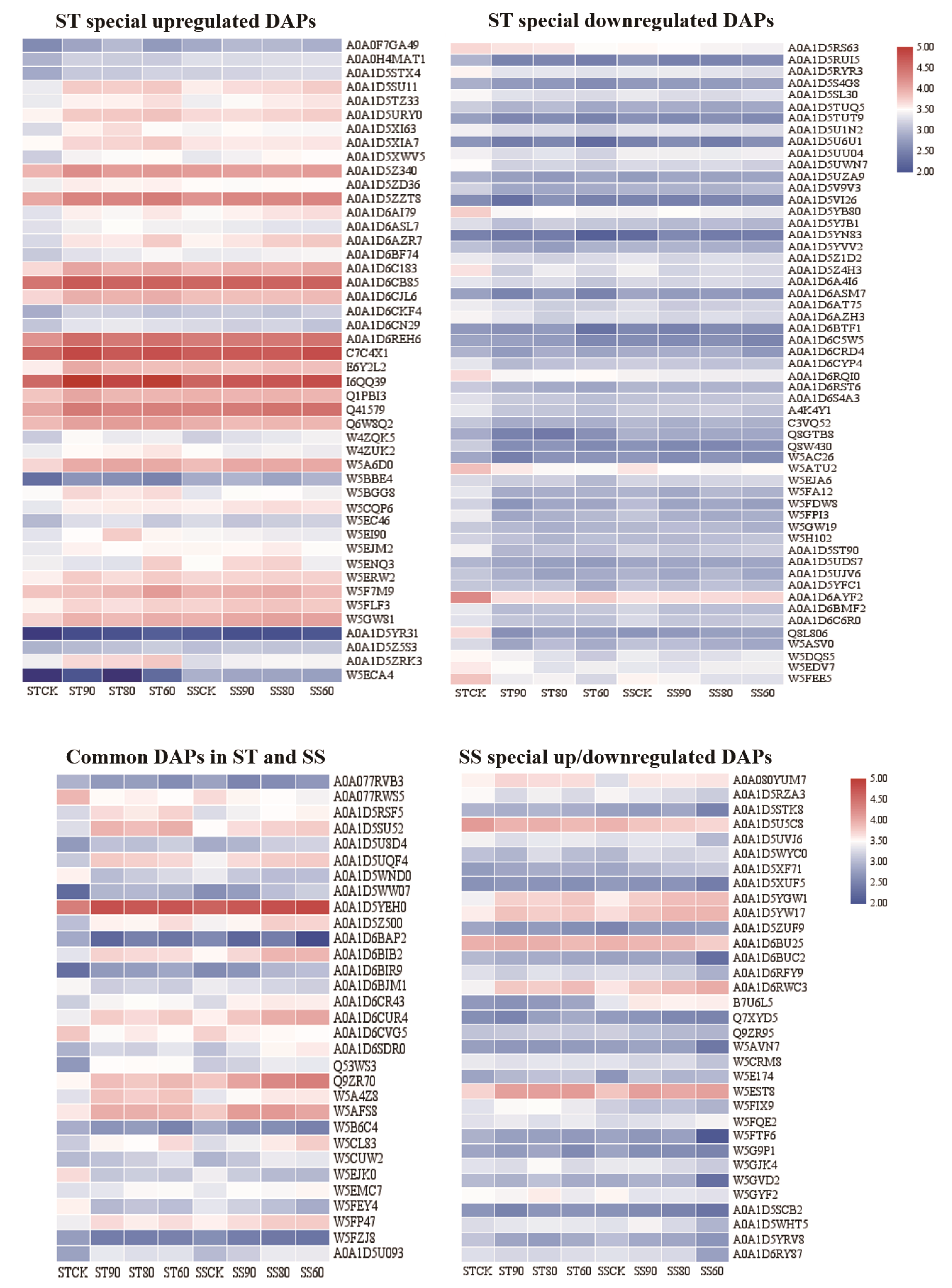

Supplement: Supplementary file 1 [file Image_1.TIF]
